# Exact biclustering algorithm for the analysis of large gene expression data sets

**DOI:** 10.1186/1471-2105-13-S18-A10

**Published:** 2012-12-14

**Authors:** Oliver Voggenreiter, Stefan Bleuler, Wilhelm Gruissem

**Affiliations:** 1 Department of Biology, ETH Zürich, Zürich, Switzerland; 2 Nebion AG, Zürich, Switzerland

## Background

Biclustering of gene expression data is used to discover groups of genes that are co-expressed over a subset of tested conditions. The objective is to maximize the detection of significant biclusters; to do so, most approaches employ a heuristic approximation in order to avoid a non-polynomial computational complexity.

Previous algorithms have focused on enabling the discovery of biologically relevant results within the scope of single studies, where data size and complexity are limited. New methods and algorithms are required in order to enable applications of biclustering to larger scale data sets that can span multiple experiments and that are potentially far more heterogenous.

## Results

The BiMax [1] algorithm uses a binary representation of the gene expression matrix that has been proven to discover enriched modules of biologically relevant genes in gene expression data. This model of biclustering allows for exact solutions, however, the BiMax algorithm performs best on a restricted size of input data. We can view the biclustering formulation of BiMax as the search for all maximal bicliques in a bipartite graph; where the nodes are genes or experiments and a connection between a gene and an experiment exists if the gene was significantly expressed in that experiment. We propose a new algorithm capable of enumerating all biclusters on such a graph. In order to solve the maximal biclique enumeration problem, we make use of the backtracking Bron-Kerbosch algorithm [2] for maximal clique enumeration. We have developed and successfully tested a new algorithm, the Bipartite Bron-Kerbosch algorithm, which uses similar principles to Bron-Kerbosch but traverses the bicliques on bipartite graphs. This approach enables the algorithm to explore all maximal bicliques without visiting branches of the search tree that contain previously discovered biclusters.

## Conclusions

Our results, see Table 1, conclude that the new algorithm is significantly faster at bicluster exploration than BiMax, demonstrating a factor *n* improvement in running time (where *n* is proportional to the input data size). For instance, with input data of 800 genes and 800 experiments, BiMax solved for the over 500 thousand biclusters in just over three minutes whereas the Bipartite Bron-Kerbosch algorithm takes approximately 3 seconds.

**Table 1:** BiMax vs. Bipartite Bron-Kerbosch Running Times. Running times of the Bipartite Bron-Kerbosch (BBK) algorithm compared to BiMax on binary matrices derived from *A. Thaliana* gene expression data. Each matrix had a density of around 12% and the algorithms were given a maximum of 1 hour to complete on the same computer. The number of biclusters in each matrix is listed in the last column.

Dimensions	BiMax	BBK	Biclusters
100x100	75msec	69msec	885
200x200	340msec	168msec	4327
400x400	3sec	354msec	37583
800x800	3min9sec	3sec	590406
1000x1000	26mins40sec	15sec	3103939
1200x1200	-	1min41sec	16118494
